# Pathophysiology, treatment, and animal and cellular models of human ischemic stroke

**DOI:** 10.1186/1750-1326-6-11

**Published:** 2011-01-25

**Authors:** Trent M Woodruff, John Thundyil, Sung-Chun Tang, Christopher G Sobey, Stephen M Taylor, Thiruma V Arumugam

**Affiliations:** 1 School of Biomedical Sciences, University of Queensland, Brisbane, Queensland 4072, Australia; 2 Department of Neurology and Stroke Center, National Taiwan University Hospital and National Taiwan University College of Medicine, Taipei, Taiwan; 3 Department of Pharmacology, Monash University, Clayton, Victoria 3800, Australia

## Abstract

Stroke is the world's second leading cause of mortality, with a high incidence of severe morbidity in surviving victims. There are currently relatively few treatment options available to minimize tissue death following a stroke. As such, there is a pressing need to explore, at a molecular, cellular, tissue, and whole body level, the mechanisms leading to damage and death of CNS tissue following an ischemic brain event. This review explores the etiology and pathogenesis of ischemic stroke, and provides a general model of such. The pathophysiology of cerebral ischemic injury is explained, and experimental animal models of global and focal ischemic stroke, and *in vitro *cellular stroke models, are described in detail along with experimental strategies to analyze the injuries. In particular, the technical aspects of these stroke models are assessed and critically evaluated, along with detailed descriptions of the current best-practice murine models of ischemic stroke. Finally, we review preclinical studies using different strategies in experimental models, followed by an evaluation of results of recent, and failed attempts of neuroprotection in human clinical trials. We also explore new and emerging approaches for the prevention and treatment of stroke. In this regard, we note that single-target drug therapies for stroke therapy, have thus far universally failed in clinical trials. The need to investigate new targets for stroke treatments, which have pleiotropic therapeutic effects in the brain, is explored as an alternate strategy, and some such possible targets are elaborated. Developing therapeutic treatments for ischemic stroke is an intrinsically difficult endeavour. The heterogeneity of the causes, the anatomical complexity of the brain, and the practicalities of the victim receiving both timely and effective treatment, conspire against developing effective drug therapies. This should in no way be a disincentive to research, but instead, a clarion call to intensify efforts to ameliorate suffering and death from this common health catastrophe. This review aims to summarize both the present experimental and clinical state-of-the art, and to guide future research directions.

## Introduction

Stroke is the world's second leading cause of mortality, resulting around 6,000,000 deaths annually [1]. It is estimated that the lifetime risk for stroke is between 8% and 10% [2]. Pathogenically, stroke involves a heterogeneous group of processes. Vessel occlusions (ischemic stroke) account for 85% of all strokes, while primary intracerebral bleeding (hemorrhagic stroke) accounts for the remainder [3]. Embolisms cause approximately 75% of all cerebral vessel occlusions, and are the most frequent cause of focally-obstructed blood flow within the brain [4]. Ischemia is defined as a reduction in blood flow sufficient to alter normal cellular function. Brain tissue is exquisitely sensitive to ischemia, such that even brief ischemic periods to neurons can initiate a complex sequence of events that ultimately may culminate in cellular death. Different brain regions have varying thresholds for ischemic cell damage, with white matter being more resilient than gray matter [5]. In addition, certain populations of neurons are selectively more vulnerable to ischemia; for example, in the hippocampus, CA1 pyramidal neurons are highly susceptible to ischemia, whereas dentate granule neurons are more resistant [5].

Early restoration of blood flow remains the treatment of choice for limiting brain injury following stroke. Improved educational efforts that emphasize recognition of the early signs and symptoms of stroke, coupled with the widespread application of thrombolytic therapy to patients with acute ischemic stroke, has decreased morbidity and mortality following stroke [6,7]. While reperfusion of the ischemic brain is clearly desirable, tissue damage often results from both the transient ischemic insult and the reperfusion process; the latter process inducing an inflammatory response that causes additional injury to the cerebral microcirculation and adjacent brain tissue [8]. Hence, a rapidly evolving area of emphasis in stroke research involves defining the molecular and cellular basis for the augmented tissue injury and inflammation associated with transient cerebral ischemia. Clinical evidence suggests that the majority of stroke patients exhibit a slow evolution of brain injury that occurs over a period of hours-to-days following the attack. This window of opportunity, limited though it is, is sufficient to provide a clinically practical target period for therapeutic intervention, with the ultimate goal of inhibiting the progression of tissue damage that normally results from both ischemia and reperfusion [9]. There are two major categories of experimental brain ischemia; namely, global and focal ischemia. In global-ischemia models, typically, two or four cervical vessels are temporarily interrupted and circulation restored after some delay [10,11]. In focal-ischemia models, the middle cerebral artery is typically occluded, either permanently or temporarily, to allow reperfusion [12,13]. We performed a comprehensive search of the scientific literature on the pathophysiology of ischemic stroke, experimental *in vivo *and *in vitro *stroke models, drug development and preclinical studies and clinical trials in ischemic stroke. A literature search for relevant articles was conducted using Pubmed, Web of Science, NIH Stroke Trials Registry and Google Scholar search. Articles were included if they met the following inclusionary criteria: (a) written in English, (b) published in a peer-reviewed journal, (c) primarily focused on ischemic stroke, and (d) primarily used quantitative research methods.

This review considers the etiology and pathogenesis of ischemic stroke, and provides a model for each facet. The pathophysiology of cerebral ischemic injury is elaborated, and experimental animal models of global and focal ischemic stroke, as well as *in vitro *stroke models, are illustrated in detail, with the different experimental strategies to analyze the injuries, explained. Finally, we analyse previous preclinical studies that used differing strategies in experimental models, as well as the results of human neuroprotection and revascularization trials, and, finally, emerging approaches for the prevention and treatment of stroke.

### Ischemic Stroke Pathophysiology

**Figure 1:**
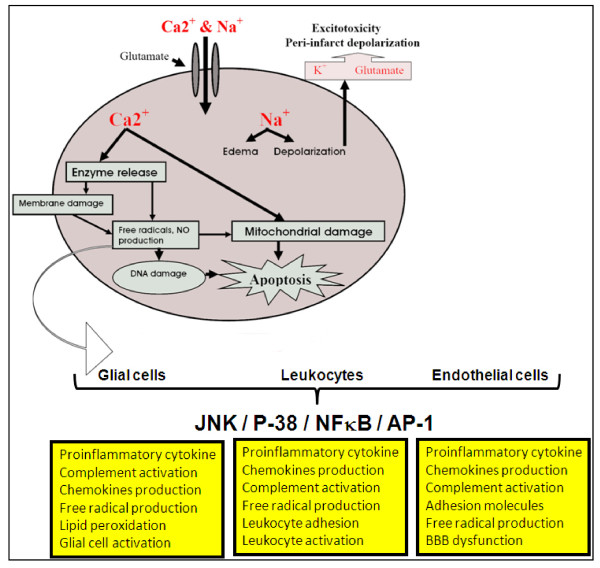
**Major cellular patho-physiological mechanisms of ischemic stroke**. Ischemia-induced energy failure leads to the depolarization of neurons. Activation of specific glutamate receptors dramatically increases intracellular Ca^2+^, and Na^+^, and K^+ ^is released into the extracellular space. Edema results from water shifts to the intracellular space. Increased levels of intracellular messenger Ca^2+ ^activates proteases, lipases and endonucleases. Free radicals are generated which damage membranes, mitochondria and DNA, in turn triggering cell death and inducing the formation of inflammatory mediators, which then induce JNK, p-38, NFκB and AP-1 activation in glial cells, endothelial cells, and infiltrating leukocytes. This culminates in pro-inflammatory cytokine and chemokine secretion and leads to the invasion of leukocytes via up-regulation of endothelial adhesion molecules.

The pathophysiology of stroke is complex and involves numerous processes, including: energy failure, loss of cell ion homeostasis, acidosis, increased intracellular calcium levels, excitotoxicity, free radical-mediated toxicity, generation of arachidonic acid products, cytokine-mediated cytotoxicity, complement activation, disruption of the blood-brain barrier (BBB), activation of glial cells, and infiltration of leukocytes (Figure 1). These are interrelated and co-ordinated events, which can lead to ischemic necrosis, which occurs in the severely affected ischemic-core regions. Within a few minutes of a cerebral ischemia, the core of brain tissue exposed to the most dramatic blood flow reduction, is mortally injured, and subsequently undergoes necrotic cell death. This necrotic core is surrounded by a zone of less severely affected tissue which is rendered functionally silent by reduced blood flow but remains metabolically active [14,15]. Necrosis is morphologically characterized by initial cellular and organelle swelling, subsequent disruption of nuclear, organelle, and plasma membranes, disintegration of nuclear structure and cytoplasmic organelles with extrusion of cell contents into the extracellular space [14,15]. The region bordering the infarct core, known as the ischemic penumbra, comprises as much as half of the total lesion volume during the initial stages of ischemia, and represents the region in which there is opportunity for salvage via post-stroke therapy [16]. Less severe ischemia, as occurs in the penumbra region of a focal ischemic infarct, evolves more slowly, and depends on the activation of specific genes and may ultimately result in apoptosis [17-19]. Recent research has revealed that many neurons in the ischemic penumbra, or peri-infarct zone, may undergo apoptosis only after several hours or days, and thus they are potentially recoverable for some time after the onset of stroke. In contrast to necrosis, apoptosis appears to be a relatively orderly process of energy-dependent programmed cell death to dispose of redundant cells. Cells undergoing apoptosis are dismantled from within in an organized way that minimizes damage and disruption to neighboring cells [15]. There are two general pathways for activation of apoptosis: the intrinsic and extrinsic pathways. Over the last decade, experimental studies have provided considerable new information characterizing apoptotic processes occurring after ischemic stroke.

#### Glutamate excitotoxicity

A significant portion of ischemia-induced neuronal damage is mediated by excessive accumulation of excitatory amino acids, leading to toxic increases in intracellular calcium [20]. Although this is an intrinsic defensive response to protect against ischemia by activating a reaction to severe cell stress, paradoxically, this increase in intracellular calcium activates multiple signaling pathways, which ultimately leads to cell death. Soon after reduction or termination of cerebral blood flow, energy-dependent cellular pumps fail due to a fall in glucose-dependent ATP generation, resulting in the flow of numerous ionic species into the cell. This results in cellular swelling through osmosis and cellular depolarization. Calcium ions (Ca^2+^) enter the cell through voltage-dependent and ligand-gated ion channels, resulting in activation of a number of proteases, kinases, lipases, and endonucleases, triggering of the intrinsic apoptotic pathway and thus ending in cell death by [18,21]. Glutamate, which is the major excitatory neurotransmitter in the brain, accumulates in the extracellular space following ischemia, and activates its receptors [22]. Glutamate receptor activation induces alterations in the concentration of intracellular ions, most notably Ca^2+ ^and sodium ions (Na^+^) (Figure 1). Elevations of intracellular Na^+ ^can be detrimental to neuronal survival at earlier time points after ischemia [23]. However, experimental studies groups suggest that glutamate toxicity is primarily dependent on Ca^2+ ^influx [24,25]. Collectively, these results suggest that cellular self-harm processes exist within the brain itself, but also, that stroke-induced central nervous system (CNS) damage may be reduced by medicinal strategies to relieve the tendency of the brain to injure itself, under conditions of stroke. The inflammatory paradox of cellular self-injury is amplified by the special sensitivity of CNS neurons to sudden deprivation of oxygen and glucose; the catastrophic temporal and anatomical nature of stroke conspires with these realities to produce consequences that are difficult to treat with medicines, and thus far, this has been a challenge beyond the capacities of modern medicine.

#### Oxidative stress

Increasing evidence suggests that oxidative stress and apoptosis are closely linked phenomena in the pathophysiology of ischemic stroke. Neurons are normally exposed to a baseline level of oxidative stress from both exogenous and endogenous sources, as are all cells in the body. Free radicals are highly reactive molecules with one or more unpaired electrons. Free radicals can react with DNA, proteins, and lipids, causing varying degrees of damage and dysfunction [26,27]. Numerous experimental and clinical observations have shown increased free radical formation during all forms of stroke injury [26,28]. Free radicals, involved in stroke-induced brain injury, include superoxide anion radical, hydroxyl radical and nitric oxide (NO). The damaging effects of free radicals are normally prevented or reduced by antioxidant enzymes and free radical scavengers [29,30]. The primary source of oxygen-derived free radicals (often referred to as 'reactive oxygen species') during ischemic-stroke injury is the mitochondria, which produce superoxide anion radicals during the electron transport process [28]. Another potentially important source of superoxide in post-ischemic neurons, is the metabolism of arachidonic acid through the cyclooxygenase and lipooxygenase pathways [31,32]. Oxygen free radicals can also be generated by activated microglia and infiltrating peripheral leukocytes via the NADPH oxidase system following reperfusion of ischemic tissue [33]. This oxidation causes further tissue damage, and is thought to be an important trigger molecule for apoptosis after ischemic stroke.

NO is generated from L-arginine through one of several NO synthase (NOS) isoforms. The neuronal form (nNOS), which requires calcium/calmodulin for activation, is expressed by subpopulations of neurons throughout the brain [34]. Inducible NOS (iNOS) is expressed by inflammatory cells such as microglia and monocytes. These two isoforms are, for the most part, damaging to the brain under ischemic conditions. A third isoform found in endothelial cells (eNOS), has vasodilatory effects and is likely to play a beneficial role by improving local blood flow [35]. NMDA receptor activation has been shown to stimulate nitric oxide (NO) production by nNOS, and to possibly play a role in excitotoxic-mediated injury in ischemic stroke [**36**]. NO diffuses freely across membranes and can react with superoxide at it point of generation to produce peroxynitrite (ONOO^-^), another highly reactive oxygen species [29]. Both oxygen-derived free radicals and reactive nitrogen species are involved in activating several pathways involved in cell death following stroke, such as apoptosis and inflammation [37-39]. A reduction of oxygen supply also leads to the accumulation of lactate via anaerobic glycolysis and so to acidosis [40-43].

#### Lipid peroxidation

Besides the production of different species of oxygen radicals, acidosis also interferes with intracellular protein synthesis. Lipid peroxidation appears to play a prominent role in the pathogenesis of stroke. The mechanism, whereby membrane lipid peroxidation induces neuronal apoptosis, involves generation of an aldehyde called 4-hydroxynonenal (4-HNE), which covalently modifies membrane transporters such as the Na^+^/K^+ ^ATPase, glucose transporters and glutamate transporters, thereby impairing their function [44]. Whilst potentially damaging via their direct actions, Ca^2+ ^and free radicals can also activate neuroprotective transcription factors, including nuclear factor-κB (NF-κB), hypoxia-inducible factor 1 and interferon regulatory factor 1 [17]. Some of these transcription factors induce the expression of inflammatory cytokines (for example, IL-1, IL-6 and TNF-α) and chemokines (for example, IL-8 and MCP-1), endothelial cell adhesion molecules (for example, selectins, ICAM-1 and VCAM-1), and other proinflammatory genes (for example, interferon-inducible protein-10) [17,45].

#### Inflammation

There are several resident cell populations within brain tissue that are able to secrete proinflammatory mediators after an ischemic insult. These include endothelial cells, astrocytes, microglia and neurons. Activation of transcription factors results in increased protein levels for cytokines and increased expression of endothelial cell adhesion molecules (CAMs) in post-stroke brain tissue [46-48]. A major role in brain inflammation following stroke is attributed to microglia, especially in the penumbral region of damage [49]. Activated microglia produce numerous proinflammatory cytokines, as well as toxic metabolites and enzymes [47,50]. In addition to microglial cells, astrocytes also have an important part in stroke-induced brain inflammation. These cells can produce both proinflammatory cytokines and neuroprotective factors, such as erythropoietin, TGFβ1, and metallothionein-2 [4]. Because of the mixed nature of microglial and astrocyte products (both destructive and protective factors), the overall role of glia may differ at different time points following stroke insult, with protective or regenerative activities occurring days to weeks after the onset of ischemia [49,51]. These factors add layers of complexity, in both adducing their pathophysiological roles in stroke, and in the goal of developing new therapeutics for stroke therapy.

#### Blood Brain Barrier (BBB) dysfunction

The brain endothelium is quite distinctive compared with other organs, as evidenced by the blood brain barrier (BBB). However, it responds to stroke injury with increased permeability and diminished barrier function, along with degradation of the basal lamina of the vessel wall, as occurs as in other organs after ischemic injury [52]. Similarly, there is considerable evidence that acute ischemic stroke enhances the interactions of brain endothelium with extravascular CNS cells (astrocytes, microglia, neurons), as well as intravascular cells (platelets, leukocytes), and that these interactions contribute to the injury process [45]. The net result of all these responses to stroke is that the cerebral vasculature assumes the following phenotypes: 1) poor capillary perfusion of brain tissue, 2) pro-adhesive for circulating cells, 3) pro-inflammatory, 4) pro-thrombogenic, and 5) diminished endothelial barrier function. These changes in normal physiological functions cumulate with each of these inflammatory responses tilting in the same detrimental direction, such that the end-result is harmful to the host CNS cells and tissues. Indeed, this is the central problem facing rapid and effective treatment of stroke.

#### Leukocyte infiltration

There is a large body of evidence that implicates leukocytes in the pathogenesis of stroke injury. The contention that leukocytes mediate reperfusion-induced tissue injury and microvascular dysfunction is supported by three major lines of evidence: 1) leukocytes accumulate in post-ischemic tissues prior to the onset of tissue injury, 2) animals rendered neutropenic exhibit a diminished injury response to ischemic stroke, and 3) prevention of leukocyte-endothelial cell adhesion with monoclonal antibodies (mAbs) directed against specific leukocyte or endothelial CAMs also affords substantial protection against stroke injury [45]. Polymorphonuclear leukocytes, of which neutrophils predominate, are therefore heavily implicated in worsening stroke outcome. Neutrophils adhere to endothelial ischemic brain vasculature in the acute phase following stroke, and infiltrate into brain parenchyma [53-56]. Rodents, with reduced circulating neutrophils (through the use of antineutrophil serum), show reduced infarct volumes and improved neurological outcomes, also indicating a pathogenic role of neutrophil accumulation and activation following stroke [57,58]. In contrast, the pathophysiological significance of lymphocyte recruitment into the brain after ischemic stroke remains uncertain. However, recent studies have shown important roles for T-lymphocytes in mediating reperfusion injury in post-ischemic brain tissue [8,59]. How lymphocytes and neutrophils may interact in stroke pathophysiology is yet unknown.

### Novel injury mechanisms

Recent experimental studies have shown several novel mechanisms to also play a role in ischemic stroke-induced brain injury. Our groups have shown that a novel CNS-specific protein, Pancortin-2, interacts with the actin-modulating protein, Wiskott-Aldrich syndrome protein verprolin homologous-1 (WAVE1), and Bcl-xL, to form a mitochondrial-associated protein complex, that attenuates neuronal apoptosis during focal cerebral ischemia in adult, but not embryonic, cerebral cortical neurons [60].

We have also shown that CNS neurons expressed several Toll Like Receptors (TLRs), and that TLR-activated pro-apoptotic signaling cascade involving jun N-terminal kinase (JNK) and the transcription factor AP-1 occurs during ischemic stroke, and is TLR 2- and 4-mediated [61,62]. Other such novel receptor-mediated signaling mechanisms are activated during ischemic stroke, also include Notch and Adiponectin receptors [63-65]. We have recently shown that gamma secretase, and adiponectin-mediated signaling, through their respective cell-surface receptors; NOTCH-1 and ADR-1 (adiponectin receptor-1), exacerbated neuronal apoptosis during focal ischemic stroke, and in combined oxygen- and glucose-deprived neurons respectively [63,64].

Collectively, these results demonstrate that there are multiple pathways influencing stroke outcome, and that there are correspondingly multiple pathways yet to be explored as new potential therapeutic targets for stroke therapy. The below sections will proffer recent advances in the development of animal models of stroke: in particular, the technical aspects of current stroke models will be assessed, and the state-of-the-art, as such, critically evaluated, along with a detailed description of the methodology of the current best-practice murine models of ischemic stroke.

### Animal Models of Ischemic Stroke

Animal models have been developed that closely resemble stroke injury seen in human patients. Many experimental models are used to study this injury, and mechanisms of cell damage are determined by testing effects of different manipulations on the extent of cell stress or death in any particular model. The three main classes of *in vivo *animal stroke models are 1) global ischemia, 2) focal ischemia, and 3) hypoxia/ischemia. The last-mentioned method, in which vessel occlusion is combined with breathing a hypoxic gaseous mixture, is almost exclusively used in young animals [66,67].

Global ischemic insults are most commonly produced by multiple vessel occlusion, and less commonly by complete brain circulatory arrest. In focal ischemic stroke models, the middle cerebral artery (MCA) is the most commonly occluded vessel; the vessel can be occluded either permanently or transiently, and damage results from both ischemia and reperfusion. Collateral blood flow may play a role in reducing the initial impact of the occlusion. Intracerebral hemorrhage frequently accompanies ischemic stroke, mainly because of disruptions of the BBB [68-70]. The choice of species for studying stroke in animals is usually limited to laboratory species, such as rodents. There are many advantages to using small animals such as rats and mice in stroke models. These species are relatively inexpensive and their cranial circulatory anatomy is similar to humans [71]. It is also possible to control the severity, duration and location of the vessel occlusion. Physiological factors can be well-controlled and histopathology allows for assessment of ischemic pathogenesis and tissue infarction.

#### Global ischemic stroke model

It has been claimed that the focal stroke models are of greater relevance than other models to the typical human stroke situation. However, global ischemia of the brain is of clear clinical relevance to cardiac arrest and asphyxia in humans [12]. In addition, it should be noted that the physiological, biochemical and functional measurements made during recovery from a global model of reversible ischemia may be important in identifying the molecular and cellular mechanisms and pharmacological actions of potential neuroprotective agents. Global models of cerebral ischemia may thus be as useful as broader focal models, provided that care is taken in the interpretation of the data. The three most widely used animals in models of global ischemia are: 1) rat - four-vessel occlusion (4-VO) or two-vessel occlusion (2-VO) combined with hypotension; 2) gerbil - 2-VO; and 3) mouse - 2-VO. A 2-VO mouse model was developed for studies in transgenic mice and is now being routinely utilised [12,68,72].

#### Rat models of global stroke

##### Four-vessel occlusion (4-VO)

This model has several advantages, including ease of preparation, a high incidence of predictable ischemic neuronal damage, a low incidence of seizures, and the absence of anesthesia (i.e., at the time of carotid occlusion) [73]. This particular model has been, and still is, widely used to investigate the effectiveness of potential therapeutic agents [12]. The rat 4-VO model involves permanent coagulation of the vertebral arteries, which alone has no deleterious effects, and temporary ligation of the two common carotid arteries. Experimental results show loss of righting reflex within 15 s, and a blood flow decrease to only 3% of control values in hippocampus, striatum, and neocortex [12,74].

##### Two-vessel occlusion (2-VO)

This model is carried out under a general anesthetic and requires the administration of a muscle relaxant. It has been well-documented that a bilateral occlusion of the common carotid arteries alone is insufficient either to bring the cerebral blood flow down below the ischemic threshold, or to upset the energy state of the brain tissue to an extent sufficient to produce detectable cell death [10,75]. To produce a damaging ischemic insult, the brain blood flow has to be further reduced using hypotension at the same time as the carotid arteries are occluded. The hypotension is normally produced in one of three ways: 1) controlled exsanguination, 2) adjunct administration of peripheral vasodilators, or 3) a combination of both approaches. The ligation of both common carotid arteries along with a blood pressure reduction to 50 mm Hg causes as much, or more, damage than 4-VO. Blood flow falls to ~3-5% in the hippocampus, neocortex, and striatum. However, in some cases blood flow is reduced to ~15% of control levels [12,76].

#### Gerbil model of global ischemic stroke

This is induced by temporarily ligating the carotid arteries, with no reduction in blood pressure. Because there are no posterior communicating arteries in gerbils, this produces profound forebrain ischemia. The changes in regional CNS blood flow are similar to those in the rat models; blood flow in the cortex is ~1%, and in the hippocampus ~4% of control values [77].

#### Mouse models of global ischemic stroke

Mouse models of global stroke are similar to rat 2-VO models using bilateral occlusion of the common carotid arteries. Several studies have demonstrated mouse global ischemia models with quantitatively uniform injury to hippocampal CA1 neurons, using techniques including 2-VO and 2-VO with hypotension [78]. However, because of the variability of collateral flow via the posterior communicating artery, it has been difficult to obtain uniform injury in the CA1 region, whilst still retaining high survival and experimental success rates. One model combines basilar artery occlusion with bilateral common carotid artery occlusion (three-vessel occlusion; [79]). However, in this model the animal survival rate is low, and CA1 neuronal injury is inconsistent. Because the mouse basilar artery runs through a narrow groove in the brain stem, and is attached to the pia mater and arachnoid membrane with arachnoid trabeculae, it is technically difficult to isolate and occlude.

#### Complete global ischemia

Complete global ischemia is generally achieved by neck-cuff, cardiac arrest, or by ligating or compressing all arteries from the heart [80]. Blood flow to the whole brain is zero or <1% in these models. Due to a very high mortality associated with this model, it is not widely used.

#### Focal ischemic stroke models

Focal ischemic stroke models, whether in larger mammals such as cats, dogs or non-human primates, or in rodents, usually involve occlusion of one MCA [18,81-83]. Focal ischemia is differentiated from global ischemia in two ways. First, even at the core of the lesion, the blood flow is almost always higher than during global ischemia, so that longer insults are required to cause damage than for global ischemia. Secondly, there is a significant gradation of ischemia from the core of the lesion to its outermost boundary, and hence there are different metabolic conditions within the affected site. Because of its duration and heterogeneity, the insult is much more complex than in global ischemia, but it is an invaluable and realistic model for human stroke, and is thus widely studied.

There are two models of focal ischemic stroke, 1) transient focal ischemia and 2) permanent focal ischemia. In transient focal ischemia models, vessels are blocked for periods of up to 3 hours, followed by prolonged reperfusion; whereas, in permanent focal ischemia, the arterial blockage is maintained throughout an experiment, usually for one or more days.

##### Transient middle cerebral artery (MCA) occlusion

**Figure 2:**
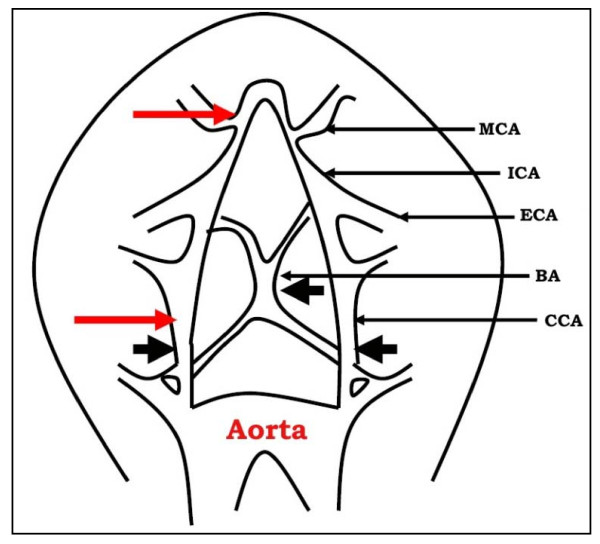
**Surgical technique for inducing global or focal cerebral ischemia in mouse**. Schematic illustration of arteries demonstrating the three points of occlusion (black arrows) for global ischemia (3-VO) and red arrows for focal ischemia. MCA: middle cerebral artery; ICA: internal carotid artery; ECA: external carotid artery; BA: basilar artery; CCA: common carotid artery.

There are two principal occlusion sites in this model (Figure 2). In proximal occlusion, the MCA is occluded close to its branching from the internal carotid artery, before the origin of the lenticulostriate arteries. A newer, and now widely used approach to proximal MCA occlusion, is the insertion of a nylon suture into the carotid artery, past the point at which the MCA branches, so that the latter is occluded at its origin.

*The procedure is as follows*: After a midline neck incision, the left external carotid artery (ECA) and pterigoparatine artery are isolated and ligated with silk thread. The internal carotid artery (ICA) is occluded at the peripheral site of the bifurcation of the ICA, and the pterigoparatine artery, with a small clip and the common carotid artery (CCA) is ligated with silk thread. The ECA is cut and a nylon monofilament, whose tip is blunted (0.20-0.22 mm for mouse) with a coagulator, is inserted into the ECA. The ECA and the inserted nylon thread are tightened with a silk suture, which prevents bleeding during advancement of the nylon thread and during its removal at the time of reperfusion, and rotated for its advancement into the ICA. After removal of the clip at the ICA, the nylon thread is advanced until light resistance is felt; the distance from the nylon thread tip to the ICA-pterygopalatine artery bifurcation is slightly more than 6 mm (mouse) and the distance to the ICA-ECA bifurcation is slightly less than 12 mm. During MCA occlusion, the parietal bone becomes pale on the occluded side and laser Doppler flowmetry reveals that blood flow in this area falls to less than 20% of baseline [84]. The nylon thread and the CCA ligature are removed after an occlusion period and reperfusion occurs with release of blood flow from ICA.

There are some potentially important artifacts with this widely-used method. Blood flow following temporary ischemia is somewhat compromised by the partial occlusion of the carotid arteries with the filament and there is quite extensive damage to small arteries in the ischemic field, including damage to endothelial and smooth muscle cells. It has been suggested that this damage may affect subsequent neuronal cell death, by exacerbating the leukocyte response in the reperfusion period [8,45,85].

##### Permanent middle cerebral artery (MCA) occlusion

The commonly-used permanent focal ischemia model involves occlusion of one or more branches of the MCA. The MCA is exposed via a trans-temporal approach. After the temporalis muscle is retracted, a 2-3 mm burr hole is drilled 2-3 mm rostral to the fusion of the zygomatic and squamosal bones. The MCA is exposed after opening and retracting the dura mater. Using a steel hook maneuvered via a micromanipulator, the MCA is elevated and electrocoagulated [86,87]. Another commonly-used permanent focal ischemia model involves occlusion of the MCA using a thread and leaving the thread in place up to 24 h [88].

##### Embolic model of focal ischemia

The procedure for this model is similar to the transient focal ischemic model [89]. Briefly, a longitudinal incision is made in the midline of the ventral cervical skin. The CCA, ICA and ECA are exposed. The distal portion of the ECA is ligated with two sutures and the ECA is cut between these two sutures. A silk suture is tied loosely around the origin of the ECA. The CCA and ICA are temporarily clamped using microvascular clips. A small puncture is made on the wall of the ECA with a pair of spring scissors. A modified PE-10 catheter connected with a PE-50 tubing (40 mm in length for 10 ml thrombus and 20 mm for 5 ml), filled with bovine thrombin is introduced into the lumen of the ECA via the puncture. The suture around the origin of the ECA is tightened and the clip on the ICA is removed. After the blood is withdrawn, the catheter is advanced up in the ICA until its tip is 1-2 mm away from the origin of the MCA. The catheter is retained there for 15 min to allow formation of a clot. Once the clot has formed, it is then gently injected into the MCA. The catheter in the ICA is removed 5 min after the clot injection and the ECA is ligated.

##### Photothrombotic distal middle cerebral artery (MCA) occlusion

The use of a photochemical reaction to produce focal cortical ischemia in the rat brain was first described in 1985 [90]. In this model, vascular thrombosis is induced by transcranial illumination with a filtered light source in combination with intravenous injection of a photosensitive dye [91,92]. Electron microscopy and light microscopy studies showed intravascular thrombotic material, red blood cell stasis, and platelet aggregates adhering to luminal surfaces inside blood vessels, with intravascular thrombosis that is responsible for the occurrence of ischemia leading to infarction. Increased permeability through disruption of the BBB by the photochemical reaction is also involved in this type of model. The method involves mounting the rat in a stereotaxic head-holder, and making a 1-1.5 cm vertical incision between the right eye and ear. With the aid of an operating microscope, a burr hole is made with a high-speed drill. Care should be taken not to injure the dura mater. The distal segment of the right MCA is thus exposed. A krypton laser operating at 568 nm (Innova 301, Coherent Inc, or 643-Y-A01, Melles Griot Inc) is used to irradiate the distal MCA at a power of 20 mW for 4 minutes. The laser beam is focused with a 30 cm focal length convex lens and positioned with a mirror onto the distal MCA. The photosensitizing dye, normally rose bengal or erythrosin B (15-25 mg/mL in 0.9% saline), is administered intravenously at a dose of 20 mg/kg over 90 seconds, starting simultaneously with 4 min of laser irradiation.

### *In vitro *models to study ischemic stroke injury

Brain slices, particularly the hippocampal slice, and primary neuronal/glial cultures from cortex, hippocampus and cerebellum of embryonic or perinatal rats and mice, have become widely used models for studying ischemia-like damage. Ischemia-like conditions are induced by replacing the normal O_2_/CO_2 _equilibrated medium with N_2_/CO_2 _equilibrated medium; typically the cultures are maintained in an incubator containing a N_2_/CO_2 _atmosphere. When glucose is maintained in the anoxic buffer, the insult is termed hypoxia, and when glucose is omitted, the insult is termed *in vitro *ischemia or oxygen/glucose deprivation (OGD). Glucose deprivation (GD) alone can also induce neuronal death with some features similar to those observed in the *in vivo *ischemia models [93,94]. Hypoxia can also be induced by treatment with cyanide (NaCN or KCN) or by incubating in an anoxic atmosphere. Chemical hypoxia results in more free radical generation than does anoxia [63]. *In vitro *models differ from *in vivo *stroke models in several aspects. Typically, a rather longer duration of the anoxic or hypoxic insult is required to kill neurons *in vitro*. ATP depletion is less severe and the release of glutamate is delayed compared to ischemia *in vivo*. The absence of blood vessels and blood flow *in vitro *eliminates important structural and functional components of the damage process present *in vivo*, including the infiltration of inflammatory cells. In addition, the composition and responsiveness of glial cells *in vitro *differs from that in the intact brain [50,95,96].

Despite these obvious differences between the two experimental systems, there are surprisingly pleasing similarities in the way isolated cells behave under conditions of substrate stress, and the way the same cells behave under the catastrophic conditions of stroke, *in vivo *[84]. Thus, we have a simple, highly controlled experimental system providing detailed basic information as to how that cell type responds to oxygen and glucose deprivation; on the other hand, the reality of the complexity of the architecture of the brain also demands a model of ischemic stroke *in vivo*. That these two approaches complement one another so well is an unusual feature in animal models of disease, and this happy congruence can only benefit research in this area.

#### Organotypic brain slice culture

Another *in vitro *approach of studying neurons in the CNS are organotypic brain slice culture techniques. These have many advantages as the neuronal morphology, cellular and anatomical relations and network connections are maintained in these types of cultures [97-99]. Organotypic slice cultures of the brain have been used increasingly to examine neuronal cell death, mechanisms of cell migration, myelination, electrophysiological activities and synapse plasticity. They have also been used to investigate basic cellular mechanisms and treatment strategies for ischemic stroke [100-103]. The principle of this approach is based on *ex vivo *cultures derived from different anatomical regions of the CNS [104], and the donor sources most commonly used are rats and mice.

Most organotypic brain slice cultures are obtained from neonatal (P0-P10) animals. The two main culturing methods for slice cultures include: the roller drum technique introduced by Gähwiler [105], and the interface cultures developed by Stoppini [106]. Slices maintained in stationary culture with the interface method are ideally suited for three-dimensional structure studies, whereas those cultured in roller tubes are often employed for imaging experiments. In OGD studies using these cultures, the cell death and apoptotic changes in neurons can be studied by looking at cellular uptake of propidium iodide and Fluro-jade staining, TUNEL staining and immunofluorescent staining for cleaved caspase-3, respectively. With growing demands for working experimental models that can replace or reduce animal experiments, such cultures certainly seem to offer a lot of promise in studying ischemic stroke *in vitro*.

#### Primary Neuronal Culture

Timed pregnant female mice or rats at days fifteen to seventeen of gestation (E15-17) are normally used as a source of embryos for primary cortical neuronal collection [107]. The pregnant mice are anaesthetized and fetuses are collectively removed. The mouse fetuses are decapitated and the heads separated and collected into ice-cold HEPES-buffered (10 mM) Hanks balanced saline solution (HBBS) lacking Ca^2+ ^and Mg^2+^. The brains are then removed and placed in HBSS in petri dishes, where the meninges and blood vessels are stripped off the surface. Under sterile conditions, the cerebral hemispheres are dissected from the brains with the aid of a dissecting microscope and a trans-illumination light source. The isolated cerebral tissues are then pooled into another petri dish containing HBSS and minced into smaller pieces for enzymatic dissociation. The tissues are then transferred to sterile 15 ml tubes containing 3-5 ml of HBSS containing 0.2% trypsin. Following a 15-20 min incubation in the trypsin solution, the tissue pieces are rinsed with fresh HBSS and incubated for 5 min in 0.1% soybean trypsin inhibitor to halt the protease reaction. Following another wash in HBBS, cells are further dissociated by triturating the tissue, using a narrowed bore sterile pasteur pipette, until most of the visible-sized tissue pieces have been disrupted. Aliquots of cell suspension are added to growth substrate-coated cell culture dishes containing Neurobasal medium with B-27 supplement (Invitrogen, USA). The culture plate-coating substrates that are known to work well for primary neuronal culture are poly-L-lysine, poly-D-lysine and polyethylene imine (PEI) [107,108]. The choice of substrate depends on the nature of experiment [107]. After an overnight coating of the plates at room temperature, the dishes are washed 2-3 times with phosphate buffered saline (PBS) and allowed to dry. The dishes are sterilized by exposure to UV light for 10 min and culture medium is added to the dishes. Cells are allowed to attach to the substrate-coated dishes for 3-6 hours and maintained in a humidified incubator (37°C with air and 6% CO_2_). The cell culture medium is then replaced with fresh medium a few hours later. Generally the neurons are allowed to mature over 5-7 days before experimentation.

#### Primary Glial Culture

Astrocytes account for about 50% of the cell population in adult mammalian brains. In addition to providing structural, metabolic and trophic support to neurons, their role in protecting neurons from injurious stimuli is slowly being unraveled [109-111]. Consequently, studying them under *in vitro *conditions is cardinal to understanding their role in stroke pathology. Astrocyte cell cultures are normally obtained from one to three day postnatal (P1-P3) mouse/rat pups. The heads are removed from pups and placed in a Petri dish with ice-cold HBSS containing 2% sucrose. The meninges are then stripped off and the mice cerebra are dissected aseptically from the residual parts of the brain. The cerebra are then cut carefully into small pieces. The tissue pieces are trypsinised following a 10 min incubation with 0.25% trypsin. Trypsin inactivation is achieved by adding Dulbecco's Modified Eagle Medium (DMEM) containing 10% fetal bovine serum and an antibiotics mix of penicillin (100 IU/ml), streptomycin (100 mg/ml) and amphotericin (0.25 mg/ml). The cerebra are dissociated by passing them through a fire-polished Pasteur pipette and the resultant cell suspension is allowed to settle for about 5 min, to allow for decantation of the large, uncleaved cell clusters. The supernatant is then centrifuged for about 5 min at 1500-2000 rpm at room temperature. The cell pellet is then dispersed in the new volume of the medium and plated on either T-75 or T-25 culture flasks at a density of about 5 million cells/ml. Following the initial seeding of cells, the medium is replaced once every 3 days. The resulting culture obtained is a mixed culture of astrocytes, microglia and oligodendrocytes. Microglia are microscopically visible on days 6-7 of the culture, remaining suspended in media as well as adhered to the bed layer of astrocytes. The remaining microglia and oligodendrocytes are separated from the bed layer of astrocytes by shaking the culture flasks on a rotary shaker placed in the incubator for at least 10 hours [112] and the media from this flask is then harvested to obtain pure microglial cultures. The remaining undetached cells are relatively pure astrocytes, and are detached following trypsin incubation for about 10 min. After addition of an equal amount of the new growth medium, the cells are centrifuged and the cell pellet is re-dispersed by gentle pipetting and seeded onto new culture flasks. Following a 3 hour incubation, this medium is aspirated out and new volume (about 10 mL) of growth medium is added. This applied procedure allows for a high purity of astrocytes to be obtained with routine culturing [113].

#### Glucose deprivation (GD) or oxygen/glucose deprivation (OGD) experiments

For GD, cultured neurons are incubated in glucose-free Locke's medium, pH 7.2, supplemented with gentamycin (5 mg/L) for 6, 12 or 24 hours. In order to mimic *in vitro*, transient focal ischemic strokes that occur *in vivo*, the neuronal cultures are exposed to OGD. The original glucose-containing neurobasal medium is replaced with a glucose-free Locke's buffer containing no serum. All media changes are followed by washes with sterile PBS (pH 7.4). Following this, the cultures dishes are exposed to hypoxia (PO_2 _<50 mm Hg) by placing them in a small, 3 L, airtight experimental hypoxia chamber (Billups-Rothenberg, San Diego, CA) with inflow and outflow connectors and circulating a hypoxic gas mixture of 95% N_2_/5% CO_2 _mixture for 15-20 min [84]. Cultures are exposed to conditions of OGD for 1, 3, 6, 12 or 24 hrs.

### Measurement of ischemic stroke damage in animal models

The measurement of dynamic changes in the ischemic brain has attracted growing attention. Ischemic brain injury in both focal and global ischemia models evolves as a progressive sequence of cellular and molecular events [17]. In this section we describe methods for analyzing brain injury and dynamic changes in the brain during ischemia and reperfusion.

#### Cerebral blood flow analysis and physiological variables

Regional microvascular tissue perfusion (cerebral blood flow) is normally monitored before, during, and after focal ischemia, using laser Doppler flowmetry. The values during ischemia are calculated as a percentage of the pre-occlusion level. The region of measurement is set at 1 mm rostral and 1 mm dorsal to the cross-over point of the left MCA and rhinal fissure, which is in the ischemic penumbra of the ischemic lesion [114]. Blood pressure, rectal temperature, and blood gases are measured during the operation in rats and mice. Normally, under controlled conditions using heating pads, there are no significant differences in temperature or blood pressure monitored at pre-, intra-, and post-ischemic time points in most studies. However, measuring and maintaining these parameters is important for proper interpretation of the technical procedure causing stroke, outcomes among animals, as well as for evaluating adhering leukocytes, which is accomplished by intravital video microscopy [115]. An estimate of shear rate in venules is obtained by fluorescence microscopy based on image analysis determinations of the maximal velocity of fluorescently-labeled red blood cells or platelets within the venules under study [116,117]. Such estimates of pseudo-shear rate in venules are obtained using measurements of venular diameter (Dv) and the maximal velocity of flowing platelets (Vplt) according to the formula: pseudo-shear rate = (Vplt/1.6)/Dv × 8 [116].

#### Quantification of cerebral infarction

**Figure 3:**
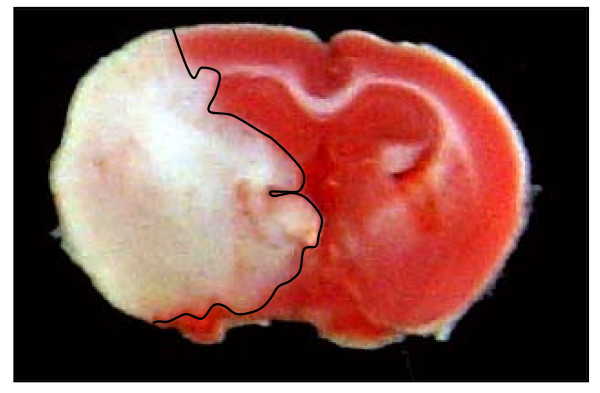
**Representative coronal brain section from a mouse that had been subjected to MCA occlusion-reperfusion**. This mouse had a one hour MCA occlusion and 72 hours reperfusion. The *red staining *indicates healthy brain tissue and the *white *indicates damaged tissue.

The size of the brain infarct in focal cerebral ischemia increases during the period of reperfusion (Figure 3). This has been shown in animal models of stroke and in human stroke patients [118]. The infarct volume is normally analyzed after 12-24 hours in transient and permanent focal ischemia models. The brain is removed and coronal sections are cut (2 mm-thick slices in rats or 1-2 mm thick slices in mice) through the entire rostro-caudal extent of the cerebral cortex. The slices are immersed in a 2% solution of 2,3,5-triphenyltetrazolium chloride (TTC). An edema index is calculated by dividing the total volume of the hemisphere ipsilateral to the MCA occlusion by the total volume of the contralateral hemisphere. An infarction index, the actual infarcted lesion size adjusted for edema, is then calculated for each animal [119].

#### Neurological assessment

The functional consequences of focal ischemic stroke injury are evaluated using a 5-point scale neurological deficit score (0, no deficit; 1, failure to extend right paw; 2, circling to the right; 3, falling to the right; and 4, unable to walk spontaneously) [120]. More recently a 14 point neurological scoring system was developed [121]. This new scoring method includes the results of motor, reflex and balance tests; a single point is awarded for the inability to perform the test or for the lack of a tested reflex.

#### Blood-brain barrier function

Albumin leakage, a quantitative index of endothelial barrier dysfunction, can be applied to the brain for determination of BBB integrity. Albumin extravasation in the brain can be quantified either by fluorescence imaging of the leakage of fluorescein isothiocyanate-labeled albumin, or sulforhodamine (Texas Red)-labelled albumin from cerebral venules, or from the clearance of the same fluorochromes from blood to the artificial CSF perfusing the brain surface [122,123]. With this imaging approach, fluorescently tagged albumin is administered intravenously to the animals 15 minutes before the baseline observation period and fluorescence intensity is detected using a silicon-intensified target camera. The fluorescence intensities within a specified segment of cerebral venules within the cranial window and in a contiguous area of perivenular interstitium are measured at various times after administration of fluorescent-albumin using a computer assisted digital imaging processor (NIH Image 1.61 on a Macintosh computer). Vascular albumin leakage is determined from the difference in fluorescence intensity between the outside and inside of the venular segment.

#### Brain edema measurement

The brains are immediately removed and divided into contralateral and ipsilateral hemispheres. The tissue samples are weighed on an electronic analytical balance to the nearest 0.1 mg to obtain the wet weight. The tissue is then dried at 90-100°C for 24 hours to determine the dry weight. Brain water content (%) is calculated as {(wet weight-dry weight)/wet weight} × 100.

#### Assessment of leukocyte and platelet adhesion

**Figure 4:**
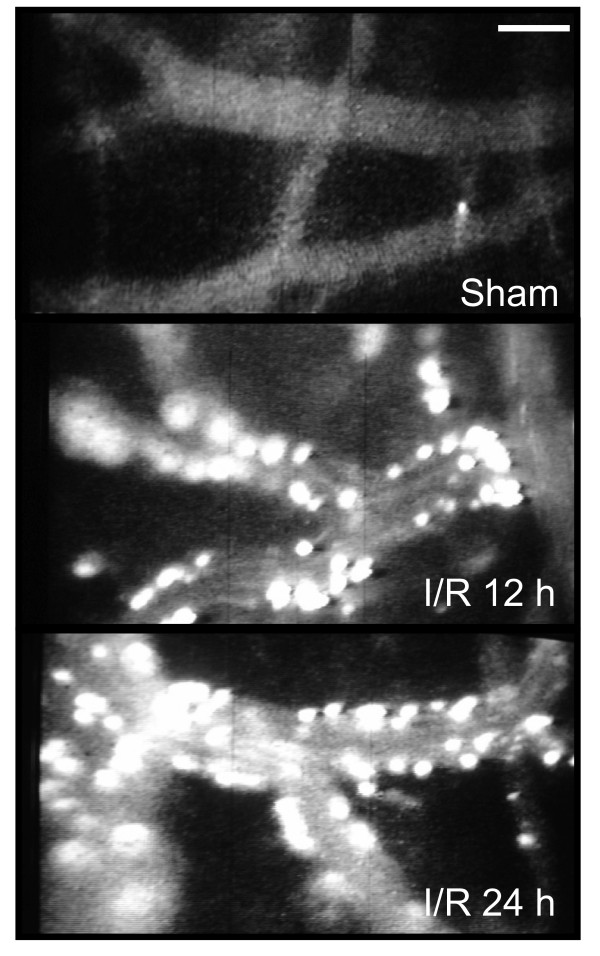
**Representative images of leukocyte interactions in pial microcirculation of a sham-operated mouse and in mice which underwent 1-hour MCA occlusion and 12 or 24-hour reperfusion**. Sham-operated animals show minimal leukocyte adhesion. Scale bar 50 μm.

Adhesion of leukocytes and platelets following ischemia and reperfusion can be monitored *in vivo *using intravital video microscopy (Figure 4). The head of the animal is immobilized and a hole drilled through the skull using a high-speed micro drill (1 mm posterior from bregma and 4 mm lateral from the midline). The dura mater is not cut because the fluorescently labeled blood cells are easily observed and intracranial pressure is well-maintained with the dura mater intact. Artificial cerebrospinal fluid is placed on the exposed brain tissue. For focal ischemia models, the observation area includes the infarcted region following MCA occlusion [124]. For global ischemia models, the observation area includes the entire cerebral cortex. Platelets are isolated from a donor animal using a series of centrifugation steps and labeled *ex vivo *with carboxyfluorescein diacetate succinimidyl ester. Once the platelet data are collected, endogenous leukocytes are labeled *in vivo *by infusing rhodamine-6G (100 μl; 0.02%) over 5 min and allowed to circulate an additional 5 min before observation. An upright Nikon microscope equipped with a SIT camera (C2400-08; Hamamatsu Photonics) and a mercury lamp is used to observe the cerebral microcirculation. The images are received by a CCD video camera and recorded on a video recorder equipped with a time-date generator (WJ-810; Panasonic).

#### Quantification of cell activation and adhesion molecule expression

The dual radiolabelled monoclonal antibody (mAb) technique is used to quantify the expression of different endothelial cell adhesion molecules, including P-selectin, ICAM-1 and VCAM-1, in the microvasculature of the brain. This method determines the relative accumulation, in any regional vascular bed, of a binding mAb to a specific endothelial surface epitope (eg, P-selectin) and an isotype-matched non-binding mAb, the latter of which is used to compensate for non-specific accumulation of the binding mAb. Different endothelial cellular adhesion molecules in the brain can be analyzed following ischemic stroke. However, adhesion molecules specifically expressed by immune cells such as LFA-1 are analyzed at least 24 hours after reperfusion. Activation of microglial cells following ischemia or reperfusion is normally analyzed using CD11b (Mac-1/CR3) antibodies.

#### Magnetic resonance imaging (MRI)

With different MRI techniques, *in vivo *diagnostic and prognostic information can be obtained on edema formation, hemodynamics, tissue structure, neuronal activation, cell migration, gene expression and more. In addition, MRI can be combined with other imaging modalities such as positron emission tomography or optical imaging to obtain complementary or supplementary information. Advances in magnetic resonance technology, such as magnets with higher field strength, more powerful gradient systems and increasing availability of targeted magnetic resonance contrast agents, allow MRI research in animal models of ischemic and hemorrhagic stroke with higher sensitivity, faster acquisition, and improved specificity (see [125] for review).

#### Measurement of protein and mRNA levels in the ischemic brain

Identification and quantification of specific proteins and mRNAs *in situ *and in brain tissue samples can provide valuable information to elucidate the signaling pathways and molecular mechanisms involved in ischemic brain damage and recovery from stroke. Proteins can be identified *en masse *by proteomic methods and individual proteins can be quantified by immunoblot or enzyme-linked immunosorbent assays [126,127]. Specific mRNAs are detected and quantified by polymerase chain reaction (PCR)-based gene array methods and by real-time PCR (see [128] for review).

### Measurement of damage in *in vitro *ischemic stroke models

#### Trypan blue exclusion test of cell viability

**Figure 5:**
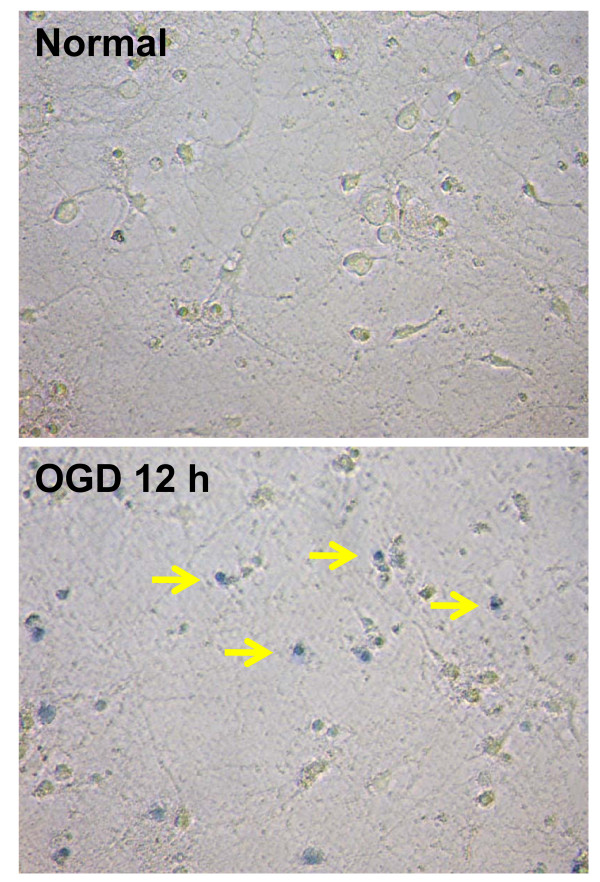
**Representative images of neuronal cell death following 12 h oxygen and glucose deprivation (OGD)**. Dead cells have altered membrane permeability, thereby facilitating the entry of trypan blue (yellow arrow) into the cell and staining the cytoplasm blue, whereas the live cells have a clear cytoplasm.

This test is based on the principle that live cells possess intact cell membranes that exclude certain dyes, such as trypan blue, whereas dead cells do not possess this ability. Hence, following OGD/GD conditions the dead cells would have altered membrane permeability, thereby facilitating the entry of this dye into the cell and staining the cytoplasm blue, and the live cells would have a clear cytoplasm (Figure 5). This test is performed by adding trypan blue into control normoxic neuronal plates containing NB medium or OGD/GD- exposed neuron plates containing glucose deprived-Locke's Buffer. Following 3-5 min incubation with trypan blue, the cells in the culture plates are fixed with 4% buffered formaldehyde and counted under a normal light microscope. In each field, the dead and total number of cells is counted and their ratio provides an estimate of percentage cell death [129].

#### MTT-assay

Cell viability can also be assessed colorimetrically using the 3-(4, 5-dimethylthiazol-2-yl)-2,5 -diphenyl tetrazolium bromide (MTT) assay [130,131]. MTT is added to the cultures and incubated at 37°C for 40 min. Following this incubation, the media is aspirated and DMSO is used to solubilise the blue formazan product. The cell plates are then assessed using a spectrophotometer at 570 nm and the live cell percentage can be compared between control normoxic and OGD-exposed cell plates.

#### Fluorescent analysis of necrosis and apoptosis

Cells undergoing apoptosis or necrosis following OGD conditions *in vitro *can be visualised using fluorescent stains like propidium podide (PI) and Hoechst 33258. Similar to trypan blue, PI is taken up only by dead cells. Hoechst 33258, on the other hand, is a nuclear stain and stains all nuclei of both viable and dead cells. This method is carried out by adding PI (10 μM) to the neuronal cultures 6-12 hours after OGD. The cells are then fixed in ice-cold 4% buffered formaldehyde. Hoechst staining is carried out post-fixation by incubating the cultures with Hoechst 33258 (1 μg/mL) for 10 min at room temperature. The cells are then analyzed under a nonconfocal fluorescent microscope. The results are interpreted as follows: the nuclei of viable cells are blue- intact Hoechst positive and PI-negative, the nuclei of apoptotic cells are red- PI-positive and fragmented (or condensed), and the nuclei of necrotic cells are red- PI-positive and round [132]. Both the apoptotic and necrotic cells also stain blue Hoechst-positive.

### Current therapeutics and preclinical studies

The fundamental question in translating results of animal experiments to clinical use is whether or not the pathophysiologic processes in animal models are relevant to those in human disease. Queries may arise regarding differences in anatomy and physiology, the pathophysiological response to injury, or between injury mechanisms in animal models and those in human disease. All of these issues are relevant and pertinent. Therapeutic manipulations generally work best when administered before, or immediately after the insult. Experiments with animal models often begin with a pretreatment protocol, to sensibly determine the proof-of-principle of the agent in the model under study. If the therapy works, it is then tested at different intervals from injury onset, to mimic clinical realities. In animal models of human stroke, most effective therapies work best within 15-30 minutes of the stroke insult; rarely are they effective after more than 3 hours from onset of injury. The most important step in limiting ischemia is the quick restoration of blood flow that occurs either naturally, or with the aid of thrombolytic drugs. Yet, this also contains the seeds of further destruction to tissues in the CNS; the reperfusion injury following an ischemic insult magnifies the clinical challenges of treating stroke. Hence, the concern is not merely related to shortening the timing of therapy after the initial insult, but also the knowledge that there are other pathological mechanisms lurking, which are often more dangerous than the precipitating events causing the stroke.

Intravenous recombinant tissue plasminogen activator (rtPA) is the first-, and only-, approved agent for the treatment of acute stroke [133]. Tissue plasminogen activator (tPA) is a serine protease that catalyzes the activation of the zymogen plasminogen by converting it to the broad-specificity, active protease, plasmin. In 1996, rtPA was approved for use in acute stroke. The NINDS trial using rtPA showed a highly significant improvement in outcome in treated patients. However, based on later examination of the outcomes of rtPA-treated patients, the benefit of rtPA was not as robust as originally predicted [134]. It has been reported that tPA can exacerbate excitotoxic neuronal death [135], suggesting an adverse effect of this drug that may, in part, counteract its clot-dissolving action.

Another treatment that shows promise is hypothermia, which may decrease metabolic demand and extend neuron survival [136,137]. Additional therapeutic approaches target the processes involved in calcium overload, glutamate excitotoxicity, oxygen radical production, apoptosis and inflammation.

#### Calcium-stabilizing agents

Cell death cascades in ischemic stroke are mediated, in part, by excessive calcium influx resulting from activation of glutamate receptors and voltage-dependent calcium channels (VDCC). In addition, the function of Ca^2+^-ATPases is compromised, resulting in prolonged elevation of the intracellular calcium concentration. Drugs that block glutamate receptors (eg, MK-801) or VDCC (eg, nimodipine and flunarazine) have proven effective in rodent models of stroke [138]. At least 14 clinical trials of nimodipine in ischemic stroke were conducted beginning in the mid 1980s. Nine trials found no effect, one trial found short-term worsened outcome with treatment, and four trials found positive outcomes [139]. Clinical trials with flunarizine found no statistically significant improvement in outcome [140]. Despite this discouraging analysis, dantrolene, which blocks ryanodine receptors, has been discussed for clinical trials as the result of beneficial effects in rodent stroke models [141].

#### Anti-excitotoxic agents

Several compounds that interfere with glutamate receptor activation have been developed and tested against experimental animal models of stroke as well as against human clinical trials. The noncompetitive NMDA antagonist MK-801 (dizocilpine) improved outcome in models of focal ischemia producing up to 75% reductions in infarct volume [142,143]. Both MK-801 and dextromorphan, another noncompetitive NMDA receptor antagonist, exhibited protective effects in experimental studies, but clinical trials were terminated early because of phencyclidine-like psychotic side effects and lack of efficacy against stroke injury [144]. Some other noncompetitive (aptiganel, ceresine,) or competitive (selfotel, eliprodil) NMDA receptor antagonists were shown to be very effective in animal stroke models, but with no significant effects in clinical trials [139]. Non-NMDA antagonists have also been developed and studied against stroke conditions. Zonampanel (YM-872) is an AMPA antagonist tested in human Phase 2 clinical trials [145]. In addition, another AMPA antagonist, SPD-502, as well as metabotropic glutamate receptor modulators, are being developed and tested against stroke injury in animals and humans [9,139]. However, the development of anti-excitotoxic agents against stroke has thus far been disappointing.

#### Antioxidants

Free radical production is enhanced in both the ischemic core and penumbra following stroke injury, and this is believed to cause much of the damage seen in these regions. There are many agents that either block free radical production or inhibit its activation that have been shown to be very effective in experimental models. Uric acid is a well-known natural antioxidant present in fluids and tissues. Administration of uric acid resulted in a large and significant reduction in ischemic damage and improved behavioral outcome [146]. Edaravone, tetramethylpyrazine, alpha-phenyl-N-tert-butyl-nitrone, FR210575 and NXY-59 are some of other free radical inhibitors that have been shown to be effective against experimental stroke injury [147,148]. Completed clinical trials with free radical scavengers, however, have had limited success after acute ischemic stroke. The free-radical-trapping agent, NXY-59, was initially reported to be efficacious in acute ischemic stroke [149], however a follow-up trial in a larger cohort of patients failed to demonstrate efficacy [150]. EGb-761 (Tanakan^®^), being developed by Ipsen, is a free radical scavenger derived from a concentrated extract of Ginkgo [151], that has recently completed a Phase 3 clinical trial with results still pending [152].

#### Anti-apoptotic agents

Accumulating evidence strongly suggests that apoptosis contributes to neuronal cell death in stroke injury. Caspases, a family of cysteine-aspartate proteases that include at least 14 members divided into three groups (I, II, and III), are essential players in apoptotic neuronal cell death [153]. Many groups have studied the effects of caspase inhibition on cerebral ischemia-induced neurodegeneration by using the broad spectrum caspase inhibitor z-VAD, either in the fluoromethylketone (fmk) or dichlorobenzoyloxopentanoic acid (dcb) form and z-DVED-fmk. Both inhibitors were neuroprotective in mouse models of transient cerebral ischemia and z-VAD was neuroprotective also in transient and permanent models in the rat [154]. Ac-YVAD-cmk (Ac-Tyr-Val-Ala-Asp-cmk), a caspase group I (caspase-1-like) inhibitor, also was shown to be neuroprotective in a mouse transient model of cerebral ischemia [154]. In addition, peptide-based caspase inhibitors have been shown to prevent neuronal loss in animal models of stroke [155]. To date however, the efficacy of anti-apoptotic agents in human stroke patients has not yet been tested.

#### Anti-inflammatory approaches

Inflammation in stroke is characterized by the accumulation of leukocytes and activation of resident microglial cells. Inflammatory cells can contribute to stroke pathology through two basic mechanisms. They form aggregates in the venules after reperfusion, or, enter infarcted tissue and exacerbate cell death through production of free radicals and cytokines [8,53]. Cell adhesion molecules such as selectins, integrins, and ICAMs permit endothelial-inflammatory cell interactions. Treatment with anti-selectin antibodies successfully decreased infarct volume by up to 70% after transient focal ischemia in mice [156]. An anti-ICAM-1 antibody has also been shown to decrease infarct size after transient, but not permanent, focal ischemia [157]. However, a recent clinical trial using the murine anti-ICAM-1 antibody enlimomab, worsened neurologic score and mortality in patients and a follow-up study using the murine anti-rat ICAM-1 antibody in rats also found an increase in infarct volume and no efficacy [158,159]. It is believed that immune activation in response to the foreign mouse protein probably accounted for the failed clinical and follow-up experimental results [159]. Recently, a Phase 2 trial using anti-CD11b/CD18 agent UK-279276, has been completed, and demonstrated that this compound is safe and well-tolerated [160]. Other targets include the mitogen activated protein kinases (MAPK), which have been linked to inflammatory cytokine production and cell death in ischemic stroke injury. SB-239063 is a MAPK inhibitor that reduced infarct size and improved neurological outcome following focal stroke in rodents, which may be an alternative target to limit inflammation in human stroke patients [161].

Matrix metalloproteinases (MMPs) are enzymes that break down components of the extracellular matrix and enhance BBB breakdown after stroke, promote hemorrhage, and increase inflammation. MMP inhibitors such as BB-94 and KB-R7785 show decreased infarct volume in treated mice after permanent focal ischemia [162]. MMP inhibitors have been evaluated in patients for their anti-angiogenic properties and are well-tolerated [163]. Although chemokines can have pro- or anti-inflammatory actions, the overall effect of chemokine up-regulation in ischemia-reperfusion injury is detrimental. NR58-3.14.3, a novel broad-spectrum inhibitor of chemokine function significantly reduced lesion volume in rats by up to 50%, and this was associated with a marked functional improvement [164]. Several other anti-inflammatory cytokine approaches were tested in experimental stroke models, including various antibodies that target inflammatory proteins. However, there have been no successful clinical trials of such anti-inflammatory agents reported so far. As mentioned earlier in this review, microglial activation following stroke insult plays a role in promoting inflammatory processes, but therapeutic approaches that specifically target microglia are currently lacking.

#### Emerging approaches for the treatment of ischemic stroke

Brain tissue injury following ischemic stroke results from the complex interplay of excitotoxicity, oxidative stress, inflammation and apoptosis. As mentioned above, two decades of basic research targeting single stroke injury mechanisms, in single-cell types, or in single-injury mechanisms in multiple cell types, have failed completely when applied in clinical trials of human strokes. On the basis of the complexity of events in cerebral ischemia and the disappointing results from human clinical stroke trials using single agent, it is unrealistic to anticipate that a single neuroprotective drug will demonstrate benefits in human stroke. Given this consistent finding, we believe that a new pleiotropic approach in stroke treatment is required, and that targeting more diverse pathogenic events in multiple cell types may prove a superior approach over the classical, single-target one.

We have recently identified Gamma-secretase inhibitors (GSIs) as a novel and potent stroke therapy [63]. Specifically, we reported that ischemic stroke can transiently activate gamma-Secretase (γ-secretase), and a single treatment with GSIs reduced ischemic brain damage and improved recovery by targeting diverse pathogenic mechanisms in multiple cell types [63]. The concept raised by our findings, that GSIs are potentially neuroprotective after stroke, was broadened by another study which showed that GSIs are beneficial for the treatment of traumatic brain injury [165].

Intravenous immunoglobulin (IVIG) is a therapeutic modality approved for the treatment of various conditions, and is increasingly used for autoimmune disorders to suppress immune-mediated tissue damage, particularly in neuro-autoimmune diseases [166]. We have recently shown that administration of IVIG to mice subjected to experimental stroke almost entirely eliminated mortality and reduced the size of brain infarction by 50-60% [84]. Moreover, not only was the infarcted area reduced, but also, within this ischemic region, neurons were spared and only occasional cell loss was observed. In additional studies we provided evidence that IVIG can directly protect neurons against ischemia-like conditions [84]. The efficacy of IVIG against stroke-induced brain injury in our study was due, in part, to its ability to selectively neutralize complement components, and by reducing cell adhesion molecule production and subsequent infiltration of inflammatory cells, and thus reducing inflammation in the infarcted region [84].

In further studies, our group provided evidence that IVIG can directly protect neurons against ischemia-like conditions. We found that OGD in cultured neurons caused an increase in levels of cleaved (enzymatically active) caspase-3 (a marker of apoptosis) and a progressive decrease in neuronal viability. Treatment with IVIG suppressed the OGD-induced increases in activated caspase-3 levels, suggesting the IVIG protects against neuronal cell death directly, by as yet unknown mechanisms [84]. In light of the extensive clinical experience with IVIG for other indications [85,166,167], our results may support consideration of the development of clinical trials to evaluate the use of IVIG in human stroke patients.

Similar to IVIG and GSIs, statins may have potential pleiotropic effects against ischemic stroke-induced brain injury [168]. Statins reduce cholesterol levels, which have been related to a reduction in vascular event risk, but they also have pleiotropic effects such as regulation of NO and glutamate metabolism, modulation of inflammatory repsonses, reduction in platelet aggregation, immune dampening activity, and anti-apoptotic effects, as well as potentially promoting angiogenesis [169-171]. Recent clinical studies suggest a neuroprotective effect of statins during the acute phase of stroke. It has been found that patients under treatment with statins prior to suffering stroke, showed higher probability of favourable outcome at three months, compared with those without previous treatment with statins, despite similar stroke severity at admission [172]. This beneficial effect is also observed in those patients who received thrombolytic therapy with rtPA [173]. Furthermore, recent prospective data indicated that the cessation of statin medication in acute ischemic stroke patients confers a significantly higher likelihood of early neurological deterioration and poorer outcomes [168]. The promising pleiotropic action of statins could also be extended to the field of neurorepair after ischemic stroke.

## Conclusions

Ischemic stroke-induced brain injury results from the interaction of complex pathophysiological processes. The molecular biology of stroke injury is a rapidly growing field of research, that may lead to the identification of novel stroke targets and directed therapies. Mechanisms of CNS cell damage are determined experimentally by testing effects of different manipulations on the extent of cell death in animal and CNS brain cells *in vitro*, and in CNS tissue slice culture models.

Several different models of stroke have been developed. The three main classes of *in vivo *animal models are global ischemia, focal ischemia, and hypoxia/ischemia. Technological advances and experimental discoveries have begun to define the cellular and molecular mechanisms involved in stroke injury. Exploration of these targets has led to the development of numerous agents that target various injury pathways. However, despite clear demonstration of numerous agents that can prevent the cascade of events leading to ischemic neuronal death in animal models, there is no obvious neuroprotective agent that has been shown to conclusively improve stroke outcome in humans. The inconsistency between animal results and clinical trials may be due to several factors including: the heterogeneity of human stroke, morphological and functional differences between the brain of humans and animals, the relatively long post-stroke delay in administration of the drugs in clinical trials, and the better experimental control of physiological variables such as temperature, blood pressure, and differences in evaluating efficacy in animal models.

The window of therapeutic opportunity in animal models is not necessarily predictive of the time window in humans, but the determination of relative windows is useful. In animal models, the time of the stroke or ischemic onset is known precisely, as is the administration of drug at precise times, whereas in humans this is less often the case. There are a number of important issues that remain unresolved regarding the translation of experimental developments to the clinical setting. Novel interventions will be required to overcome hurdles associated with bench-to-bedside translation.

We propose that a new paradigm for drug development for stroke treatment is required. Instead of focusing on single molecular targets on single cell types - which has so far been a clinical failure, we suggest that targets with more global signaling pathways and diverse cell loci, be investigated. We call this the pleiotropic approach to stroke treatment. New, broad-spectrum agents being currently investigated, such as IVIG and GSIs may prove more fruitful therapeutically in this regard.

## Competing interests

The authors declare that they have no competing interests.

## Authors' contributions

TMW, JT, SCT, CGS, SMT and TVA conceived and wrote the manuscript. All authors read and approved the final draft.
